# Older African American Women’s Mental Health and Subjective Memory: The Role of Social Determinants of Health

**DOI:** 10.1093/geroni/igaf122.4287

**Published:** 2025-12-31

**Authors:** Gail Wallace, Ian McDonough, Roland Thorpe, George Rebok

**Affiliations:** Johns Hopkins University, Baltimore, Maryland, United States; Binghamton University, Binghamton, New York, United States; Johns Hopkins Bloomberg School of Public Health, Baltimore, Maryland, United States; Johns Hopkins University, Baltimore, Maryland, United States

## Abstract

This study examined the role of psychosocial coping ability on depression and subjective memory outcomes moderated by social determinants of health in older African American women in the Advanced Cognitive Training for Independent and Vital Elderly (ACTIVE) study. Path analysis modeling was done using an autoregressive cross-lagged modeling approach on longitudinal data with 607 older African American women (Mean Age = 72.17, 65-91) residing in U.S. cities: Birmingham, Boston, Indianapolis, Baltimore, State College, PA, and Detroit as part of the ACTIVE study. Bidirectional cross-lagged effects modeled from depressive symptoms to subsequent subjective memory and vice versa, at each lagged time interval from baseline to 10 years following baseline was implemented. The social determinants of health (SDH) of study participants were divided into high and low groups via a median split and submitted to a multigroup comparison. Covariates included age, training group, booster status, and baseline physical and general health. African American women with higher levels of personal control reported higher levels of subjective memory (β = .20, SE = .038, p < .001) and fewer depressive symptoms (β = -.24, SE = .037, p < .001). Longitudinally, higher levels of personal control also were positively associated with improved subjective memory at Year 2 (β = .14, p = .004). These effects were moderated by several SDH factors. The present findings offer a window into how different types of SDHs shape dementia risk differentially through mental and cognitive health, especially in understudied older minority population demographics consisting of AA women.

